# Progress in the Application of Iron-Based Plant Derived Biochar Catalyst for Fenton-like Remediation of Organic Wastewater: A Review

**DOI:** 10.3390/molecules30234549

**Published:** 2025-11-25

**Authors:** Xiao Wang, Dongqing Zhang, Yan Cheng, Binkui Wu, Lanyi Sun

**Affiliations:** 1 State Key Laboratory of Heavy Oil Processing, College of Chemistry and Chemical Engineering, China University of Petroleum (East China), Qingdao 266580, China; b24030032@s.upc.edu.cn (X.W.); b25030033@s.upc.edu.cn (Y.C.); z24030105@s.upc.edu.cn (B.W.); 2 School of Economics, Zhejiang Gongshang University, Hangzhou 310018, China; 23010020006@pop.zjgsu.edu.cn

**Keywords:** fenton-like, iron-based, plant derived biochar, environment remediation, advanced oxidation process

## Abstract

This paper focuses on the research progress of iron-based plant-derived biochar in the field of Fenton-like organic wastewater treatment. Given the severe organic pollution problems in water bodies, advanced oxidation technologies have garnered significant attention. In Fenton-like reactions, iron-based catalysts play a crucial role but have limitations, while the characteristics of biochar make it an ideal carrier. This paper provides a detailed account of the preparation methods for iron-based plant-derived biochar, including one-step pyrolysis and co-precipitation methods, each with its own advantages and disadvantages. It introduces the application of iron-based plant-derived biochar in Fenton-like reactions, including single-metal, multi-metal, and composite material forms, and explains the activation mechanisms involving radical and non-radical pathways. Finally, it summarizes the advantages of this material and points out the need for further research in areas such as cost assessment, metal leaching, practical water body application, and intermediate product toxicity assessment, providing comprehensive references and directional guidance for future studies.

## 1. Introduction

The presence of organic pollutants in water bodies worldwide, such as personal care products, pesticides, and endocrine-disrupting chemicals, poses a serious threat to ecosystems and human health, and has become an extremely challenging issue [1]. Advanced oxidation processes (AOPs) have attracted much attention as an efficient, economical, and stable chemical remediation technology. Strong oxidizing free radicals generated in the reaction system are utilized to decompose organic pollutants into small-molecule substances, and even mineralize them into CO_2_, H_2_O, and corresponding inorganic ions to achieve complete removal of pollutants [2]. Among them, the Fenton reaction is one of the effective technologies for the oxidative decomposition of organic pollutants, which mainly relies on the highly reactive oxygen species (ROS) generated by Fe^2+^ activating hydrogen peroxide (H_2_O_2_) to oxidize and degrade organic pollutants in wastewater [3]. However, the homogeneous Fenton technology with the addition of soluble Fe^2+^ inevitably produces a large amount of iron-containing sludge, which further increases the difficulty of wastewater treatment. Thus, heterogeneous Fenton technology has gradually come into people’s sight [4].

Common oxidants in Fenton-like reaction technologies include H_2_O_2_, peroxymonosulfate (HSO_5_^−^, PMS), and persulfate (S_2_O_8_^2−^, PDS). The principle of activation of oxidants by transition metal is that transition metal ions (Me) such as Fe^2+^, Mn^2+^, and Co^2+^ transfer electrons to oxidants to generate ROS like hydroxyl radicals (^•^OH) and sulfate radicals (SO_4_^•−^) (Equations (1)–(4)). Among them, iron is the second most abundant metallic element in the Earth’s crust. Due to its low cost and environmental friendliness, it has been widely used in the field of water pollution control [5]. The performance of iron-based catalysts in Fenton-like reactions is closely related to many factors [6]. These factors include the inherent properties of the active sites, the number of active sites available for pollutant interaction, and the physical properties of the catalyst, such as specific surface area, porosity, and electrical conductivity, which also have a significant impact on the performance of the catalyst. In addition, the cyclic process between Fe^2+^/Fe^3+^ active sites and the reactions occurring on the catalyst surface play an indispensable role in increasing the rate of ROS generation and improving the overall efficiency of Fenton-like reactions [7,8]. Therefore, a systematic summary of the design concepts, preparation methods, and intrinsic relationship between the microstructure and macro performance of iron-based catalysts in Fenton-like reactions is expected to provide inspiration for the design and construction of efficient iron-based Fenton-like catalysts, thereby opening up new research perspectives and technical pathways in the field of water pollution control.

H_2_O_2_ + Me^n+^ → OH^−^ + ^•^OH + Me^(n+1)+^ (1)

HSO_5_^−^ + Me^n+^ → SO_4_^•−^ + OH^−^ + Me^(n+1)+^ (2)

HSO_5_^−^ + Me^n+^ → SO_4_^2−^ + ^•^OH + Me^(n+1)+^ (3)

S_2_O_8_^2−^ + Me^n+^ → SO_4_^•−^ + SO_4_^2−^ + Me^(n+1)+^ (4)

Over the past few decades, various types of iron-based Fenton-like catalysts have been extensively studied for their excellent potential, including zero-valent iron (ZVI), iron oxides (Fe_3_O_4_, γ-Fe_2_O_3_, and α-Fe_2_O_3_), iron oxyhydroxide (α-FeOOH), iron disulfide (FeS_2_), iron oxychloride (FeOCl), etc. [9,10,11,12,13]. These catalysts can self-regulate the pH of the solution and release Fe^2+^ in a controlled and slow manner, which helps reduce the leaching of Fe ions from the system. However, iron-based catalysts synthesized without the addition of support materials have the disadvantages of being prone to agglomeration, oxidation in air, and leaching of metal ions [9].

During the course of heterogeneous catalytic research, the metal-support interaction (MSI) effect was first discovered and defined in the late 1970s. Currently, MSI is widely recognized as a special form of interaction between metals and transition metal oxides. In order to effectively optimize the comprehensive performance of supported metal catalysts, researchers have extensively explored the synthesis methods of MSI and made significant progress in understanding its catalytic mechanism. These research findings not only deepen our understanding of the microscopic interaction mechanisms in heterogeneous catalytic systems but also provide a solid theoretical foundation and technical support for the development of high-performance supported metal catalysts, thereby significantly advancing the field of heterogeneous catalysis toward more efficient and precise directions [10,11].

In recent years, a large number of support materials with high porosity, high specific surface area, and corrosion resistance have also been developed, such as activated carbon fibers (ACFs), activated carbon (AC), biochar (BC), carbon nanotubes (CNTs), etc. [12,13,14,15]. In recent years, researchers have published several reviews on supported iron-based materials for Fenton-like degradation of pollutants. For example, Shaida et al. [16] reviewed the applications of natural iron-based materials and artificially synthesized supported iron-based materials in Fenton-like wastewater treatment. Nawaz et al. [17] described various preparation strategies of FeS-based composites and their high efficiency and stability in AOPs for organic wastewater treatment. Lu et al. [18] summarized the latest progress of iron-based biochar catalysts in AOPs remediation of emerging pollutants. Wang et al. [19] comprehensively discussed the challenges of biochar-supported iron-based catalysts in heterogeneous catalytic ozonation in water treatment technology and further explored the activation mechanism.

Biochar can be easily produced by pyrolyzing biomass feedstock under high-temperature and oxygen-deprived conditions. Due to its excellent physicochemical properties, such as high carbon content, high specific surface area, abundant surface functional groups, and well-developed pore structure, biochar has found widespread application in adsorption and catalysis fields [20]. Biomass feedstocks are widely available and are typically categorized into three types: plant-based, solid waste (such as sludge), and animal-based. Biochar produced from plant-based biomass not only reduces production costs but also enhances the biological value of plants, making it widely applied in fields such as the environment, agriculture, and energy. It represents an effective approach to resource-efficient utilization [12].

It is worth noting that although plant-based biomass is mainly composed of organic matter, the small amount of endogenous mineral components it contains play a significant catalytic role in the biomass pyrolysis process. Research has confirmed that the mineral components in plant-based biomass can participate in reactions during pyrolysis, thereby affecting the application efficiency of biochar in environmental fields such as adsorption and carbon sequestration [13].

Therefore, in this review, based on iron-based plant-derived biochar combined with Fenton-like reagents, the application of iron-based plant-derived biochar in Fenton reactions is reviewed. Meanwhile, the basic structure of biochar is introduced, with further focus on biomass raw materials and pyrolysis conditions. Although iron-based catalysts are currently a hot topic, there is a lack of systematic research on the interaction between iron and plant-derived biochar. This paper aims to present the latest review on the application of plant-derived biochar in iron-based catalysts, providing new ideas for the design of iron-based catalysts for the future application of Fenton-like technology in organic wastewater treatment.

## 2. Preparation of Iron-Based Plant-Derived Biochar

### 2.1. Biomass Sources of Plant-Derived Biochar

Plant biomass has a unique lignocellulosic structure (lignin, cellulose, and hemicellulose). Among these, cellulose serves as the core, surrounded by the tightly arranged structures of hemicellulose and lignin. The unique carbon-rich properties of lignocellulosic biomass help increase the carbon content of biochar, making it a potential carbon precursor for the preparation of porous biochar [19]. Plant-based biomass is primarily divided into herbaceous, woody, and shell types. Crop residues such as corn stover, wheat straw, and rice straw are typical examples of herbaceous biomass, characterized by their abundant quantities, widespread distribution, and high carbohydrate content. After pyrolysis, the resulting biochar exhibits a certain degree of porosity [20]. Woody plants such as oak and maple trees are rich in cellulose and lignin, which provide a rich carbon source for biochar and form structurally stable biochar during pyrolysis. Lignin helps enhance the aromatic structure of biochar, giving it a high specific surface area and porosity [21]. Additionally, shelled biomasses such as coconut shells and walnut shells have a unique structure that makes the biochar formed after pyrolysis chemically stable, with a well-developed microporous structure and high specific surface area [22]. More importantly, it has been reported that invasive plants have been selected as an unbeatable choice for biochar feedstock due to their high diversity and wide distribution, an initiative that can simultaneously achieve ecosystem protection and resource recycling [17].

Biochar is a carbon-rich porous material derived from the thermochemical conversion of biomass, and its physicochemical properties are highly sensitive to both feedstock composition and pyrolysis parameters [23,24,25,26,27]. When integrated with iron species, biochar not only provides a high-surface-area support for catalytic reactions, but also contributes surface defects, π-electron systems, and heteroatom sites that accelerate electron transfer during radical and non-radical oxidation processes [17,28]. Recent studies have shown that endogenous minerals (e.g., K, Ca, Si) in lignocellulosic biomass and exogenous dopants (e.g., N, S, P) can influence graphitization, pore formation, and Fe speciation during pyrolysis, leading to improved catalyst durability and low-pH tolerance [29,30]. Compared with commercial activated carbons, biochar’s in situ iron anchoring and structural tunability make it especially promising for scalable, low-cost advanced oxidation processes [31,32].

### 2.2. The Influence of Factors on the Performance of Biochar

**Figure 1:**
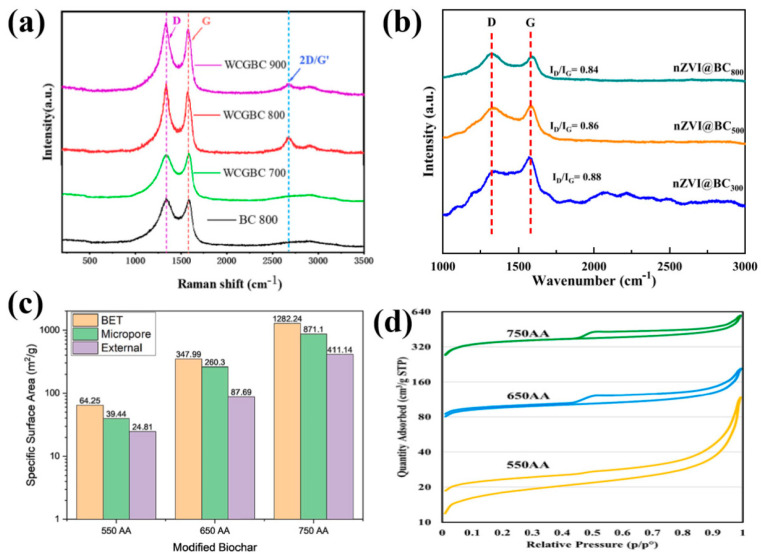
The Raman spectra (**a**,**b**), specific surface area (**c**), and N_2_ adsorption–desorption curves (**d**) of biochar obtained by heat treatment under different temperature conditions [33,34,36].

Specific surface area (SSA) and the degree of graphitization of biochar are important indicators for evaluating the performance of biochar. It is well known that the performance of biochar changes with variations in pyrolysis temperature. As the pyrolysis temperature increases, the carbon layer structure transforms from amorphous aromatic carbon to conjugated aromatic carbon and ultimately to graphitic carbon [33]. Therefore, it can be assumed that the pyrolysis temperature is decisive for the degree of graphitization of biochar. As shown in Figure 1a,b, in order to illustrate the relationship between the pyrolysis temperature and the degree of graphitization and structure of biochar materials, Wen and Qu et al. [33,34] used Raman spectroscopy (Raman) to characterize carbon materials obtained from pyrolysis treatment at different temperatures. In this case, the D band denotes sp^3^ defects such as amorphous carbon layers, disorder, edges, and boundaries of the carbon material while the G band denotes the E_2g_ vibrations of sp^2^ hybridized graphitic carbon atoms, and the I_D_/I_G_ band is used to denote the degree of graphitization of the material in the existing studies [35]. From the figure, it can be seen that the degree of graphitization of carbon materials is positively correlated with the pyrolysis temperature under certain temperature conditions. Similarly, the pyrolysis temperature affects the SSA of biochar. Rohman et al. [36] compared the SSA and pore volume of biochar at different pyrolysis temperatures (Figure 1c,d), and the BET model showed that increasing the temperature increases the porosity of the modified biochar, which leads to an increase in specific surface area. Nevertheless, the specific surface area (SSA) of raw biochar remains relatively low compared to traditional activated carbon. The lower specific surface area limits the performance of biochar in adsorption and catalysis. To expand the pores and increase the specific surface area, activation treatment is typically required, with common methods including the addition of NaOH and KOH. Additionally, heteroatoms (such as nitrogen, sulfur, phosphorus, boron, etc.) can be introduced into the biochar to prepare heteroatom-doped biochar. These heteroatoms share similarities with carbon atoms in terms of atomic radius, orbital characteristics, electronegativity, and charge density [37]. Heteroatom doping as an important strategy for the modification of carbon-based materials has many significant effects. The introduction of heteroatoms can change the charge density distribution of BCs and break the original sp^2^ hybridized orbitals of BCs, thus enhancing the electron mobility, increasing the number of defect edges, and introducing brand-new active centers, which effectively accelerates the process of electron transfer reaction on BCs. Among the many heteroatom doping strategies, nitrogen doping is one of the most effective methods to endow active sites on biochar to activate oxidants [38]. Depending on different synthesis conditions, various forms of nitrogen atoms can be introduced into biochar, including pyrrole nitrogen, pyridine nitrogen, and graphite nitrogen, and the types and contents of these nitrogen atoms can be regulated to meet different redox reaction requirements.

Researchers have also found that the modification of biochar using iron-based materials can similarly increase its specific surface area [39]. The iron element in biomass precursors alters the distribution of pyrolysis products, thereby affecting the performance of biochar. On the one hand, it can inhibit tar formation, which may promote the expansion of biochar pore structure under high-temperature conditions. On the other hand, iron-based materials have good magnetic properties, which facilitate material recycling and reduce wastewater treatment costs through material recovery. In addition, iron carbides can mediate the oxidation process of carbon matrices, ultimately helping to increase the specific surface area of biochar [40,41]. However, biochar suffers from the drawbacks of low specific surface area and unstable catalytic performance, which greatly limit its application in practical scenarios. To effectively overcome these inherent drawbacks, the introduction of iron species into the biochar system to enhance its catalytic activity may be the optimal strategy to construct efficient and stable biochar-based catalysts.

### 2.3. Strategies for Synthesizing Iron-Based Plant-Based Biochar

There are various methods for the synthesis of iron-based plant-based biochar, and currently the commonly used ones include one-step pyrolysis, co-precipitation, hydrothermal carbonization, green synthesis, ball milling, and chemical reduction. Table 1 summarizes the various synthesis methods of iron-based plant-based biochar and compares the advantages and disadvantages of iron-based plant-based biochar synthesis methods.

#### 2.3.1. One-Step Pyrolysis

**Figure 2:**
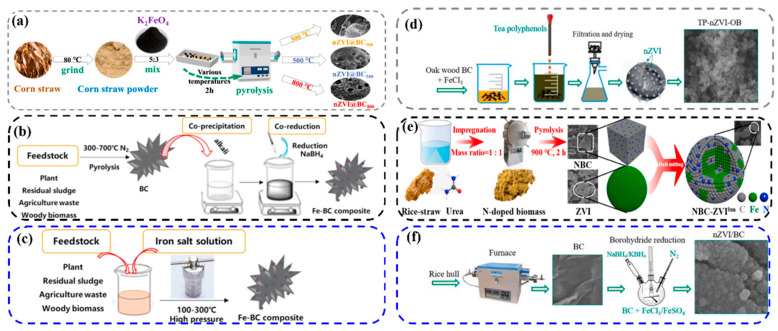
A range of methods for the preparation of iron-based biochar materials: one-step pyrolysis (**a**), co-precipitation (**b**), hydrothermal carbonization (**c**), green synthesis (**d**), ball milling (**e**), and chemical reduction (**f**) [40,41].

Pyrolysis is one of the most commonly used methods in the synthesis of iron-based biochar by virtue of its high efficiency and flexibility [42]. The specific operation process is as follows. First, the collected biomass is carefully washed in water followed by a drying process, and, after drying, it is ground into powder form and stored for reserve. Immediately afterward, the spare biomass powder was added into the iron-containing solution or iron-containing solid, and the two were fully mixed by stirring or grinding. Once the mixing is complete, the resulting precipitate is collected and dried again. Finally, the dried precipitate is calcined in air or in a specific gas atmosphere, and the iron-based biochar material is obtained (Figure 2a). Qu et al. [33] obtained nZVI@BC800 from direct pyrolysis of K_2_FeO_4_ mixed with corn stover by direct milling of the feedstock, which has a more porous structure and disorder compared to BC. Interestingly, the presence of K_2_FeO_4_ promotes the production of a large amount of CO_2_ and CO from corn stover during the pyrolysis process in order to increase the degree of disorder, whereas the corn stover is heated through carbon reduction of K_2_FeO_4_ to produce nZVI. The two feedstocks showed a strong synergistic effect. With further research, some functionalization strategies were also derived with one-step pyrolysis. For example, Li et al. [43] further modified the material obtained by mixed pyrolysis of FeSO_4_·7H_2_O and sawdust with sodium dodecyl sulfate, and the characterization revealed that the addition of FeSO_4_·7H_2_O increased the graphitization degree of the material, while the modification of SDS enhanced the hydrophobicity of the material, which in turn facilitated the material’s contact with organic pollutants

While one-step pyrolysis can produce biochar with a high surface area and significant iron content, its performance is highly dependent on both feedstock composition and pyrolysis conditions [41]. For example, the use of agricultural residues such as rice husks or corn stover may result in a lower carbon yield but can enhance the biochar’s porosity and surface area [43]. However, the non-uniform distribution of iron particles often limits the material’s effectiveness in catalytic applications, necessitating further optimization. Despite these challenges, one-step pyrolysis remains a preferred method for biochar production, particularly in applications where uniformity is not critical [44].

#### 2.3.2. Co-Precipitation

The co-precipitation method has also been applied to the preparation of iron-based biochar due to its relative ease of operation. In general, the method is divided into two steps: first, the biomass is pyrolyzed to carbonize it to form biochar, followed by impregnation by iron salts, which uses alkalis to precipitate iron onto the biochar. Wang et al. [45] used tea dregs as a raw material, and the tea dregs biochar (T-BC) obtained from pyrolysis was added to a mixture of FeSO_4_ and FeCl_3_ solution. The iron component was induced to precipitate on the surface of T-BC by dropping the NaOH solution. After sufficient mixing and aging treatment, the products were collected and dried to obtain the final Fe_3_O_4_@T-BC composite. The prepared Fe_3_O_4_@T-BC presented a rough surface with a well-developed pore structure, and Fe_3_O_4_ was uniformly dispersed on the T-BC surface. After successful loading of Fe_3_O_4_, the specific surface area of the composite increased to 97.41 m^2^/g, the total pore volume increased to 0.098 m^3^/g, and the saturated magnetization strength reached 44.64 emu/g, exhibiting excellent magnetic properties, which are favorable for recycling the material. Similarly, Tian et al. [45] successfully synthesized Fe_3_O_4_/BC composites by adding ammonia by the reverse precipitation method using corn stover biochar, FeSO_4_, and FeCl_3_ as starting materials. Fe_3_O_4_/BC composites inhibited the Fe_3_O_4_ nanoparticles agglomeration phenomenon and generated more oxygen vacancies and edge active sites, with a specific surface area as high as 72.27 m^2^/g, as well as a rich pore volume. In addition, its saturation magnetization intensity was 46.58 emu/g, exhibiting good magnetic properties.

#### 2.3.3. Hydrothermal Process

The hydrothermal method is an efficient and economical synthesis method. The hydrothermal method is a promising, cost-effective, and environmentally friendly method to produce magnetic carbonaceous materials (Figure 2c). And this technique is widely used as it eliminates the need for complex synthesis stages, extreme temperatures, high energy inputs, and harsh chemicals [46]. Saygılı et al. [47] used pomegranate seeds as a raw material and obtained Fe@PWHC with rich functional groups under optimized hydrothermal conditions (220 °C, 12 h), which achieved simultaneous carbonization and magnetization of the material. However, due to the relatively mild hydrothermal carbonization process, the conversion of biomass to biochar may not be as high as that of high-temperature pyrolysis, resulting in a low yield of the material and limiting large-scale production. Min et al. [48] prepared Fe-PP-Hy by hydrothermal synthesis using grapefruit peels as the raw material at 200 °C, but the X-ray diffraction peaks were of lower intensity, which may be attributed to the cellulose in the hydrate’s amorphous region. The specific surface areas of PP-Hy and Fe-PP-Hy were 8.13 m^2^/g and 6.13 m^2^/g, respectively, and the specific surface area of Fe-PP-Hy was reduced compared with that of PP-Hy, which indicated that the pore structure of the material was not well developed during the hydrothermal process, and it was speculated that the reason might be the limited degree of cyclization and condensation reactions during the hydrothermal period.

#### 2.3.4. Green Synthesis Process

The green synthesis method uses natural biomass and non-toxic and non-harmful iron sources to synthesize under mild conditions (e.g., ambient temperature, atmospheric pressure, or close to ambient temperature and pressure), avoiding the use of toxic and harmful chemical reagents and harsh conditions such as high temperatures and pressures. Zhao et al. [49] used corn stover biomass and natural pyrite pyrolysis to prepare FBC3 and used it for soil remediation, and the catalyst could not be separated after soil remediation due to the economic and green nature of the raw material of FBC3 and the potential environmental hazards associated with the catalyst injection in soil remediation. The catalyst could not be separated after soil remediation, the potential environmental hazards caused by catalyst injection in soil remediation were avoided. Of course, the catalyst obtained by the green synthesis method also reduces the secondary pollution caused by the material in the actual organic polluted wastewater remediation process. In addition, it has also been found that in the preparation process of zero-valent iron composite carbon materials, green plant extracts such as tea polyphenols can be used to reduce iron salts instead of chemical reductants so that they can be precipitated on the surface of the carbon materials (Figure 2d) [50].

#### 2.3.5. Ball Milling Process

Ball milling has been shown to produce composites with controllable particle sizes, which can reduce the size of BC to the nanoscale and enhance the dispersion of iron materials, as well as pyrolysis of biomass followed by ball milling in order to increase SSA and oxygen-containing functional groups and to generate new mesopores and defects (Figure 2e) [51]. Yu et al. [52] prepared pyrolyzed carbon BC and ball milled carbon MBC from pine sawdust, respectively, and by the co-precipitation method Fe_3_O_4_@BC and Fe_3_O_4_@MBC were further synthesized. Among them, MBC not only had a larger specific surface area, but also an increase in the average pore size and the formation of mesoporous structure compared with BC. Fourier transform infrared spectroscopy (FTIR) analysis showed that after ball milling treatment, the carbon content on the MBC surface increased while the oxygen content decreased. When loaded with Fe_3_O_4_, the oxygen and iron contents in Fe_3_O_4_@BC and Fe_3_O_4_@MBC increased. Raman analysis showed that the ID/IG value of MBC was 0.96, while that of BC was only 0.92, which indicated that the ball milling process induced the formation of a large number of defective sites. Zhang et al. [53] prepared ZVI/BC material using wheat straw as the raw material. After ball milling treatment, the dispersion of ZVI on the surface of biochar was significantly improved, which enhanced the exposure of active site iron. The contact between ZVI and biochar was more compact, and the specific surface area reached 3.77 times of that before ball milling, and the pore volume increased to 1.98 times of that before ball milling. These changes greatly enhanced the electron transfer ability of the material as well as its ability to remove pollutants.

#### 2.3.6. Chemical Reduction Process

Chemical reduction allows precise control of the chemical form of iron by selecting suitable reducing agents and reaction conditions, such as the reduction in iron ions to zero-valent iron or to iron compounds in specific valence states (Figure 2f). This is important for the preparation of materials with specific functionalities (*e.g.*, *zero-valent iron-based biochar with high catalytic activity*) [16]. Essentially, chemical reduction is a type of co-precipitation method. However, the chemical reduction reaction usually involves several complex chemical reaction steps, and the reaction conditions (*e.g.*, *temperature*, *pH*, *etc.*) are strictly required, and poor control may lead to incomplete reaction or generation of unwanted by-products, which may affect the quality of the materials. Moreover, the reducing agent used in the chemical reduction method may be toxic and harmful chemical reagents, such as sodium borohydride, which can be harmful to the environment and operators if not handled properly during the process [13,54]. Therefore, in recent years researchers have gradually discovered the carbothermal reduction method as well as the use of green and natural reductants instead of sodium borohydride.

In summary, there are multiple strategies for synthesizing iron-based biochar materials, each with its own unique characteristics and advantages. The one-step pyrolysis process, with its simple and direct workflow, holds potential for efficient production. Although it faces challenges such as uneven iron distribution and difficulty in controlling composition, it still demonstrates application potential in fields where material uniformity requirements are relatively low. The co-precipitation method stands out for its excellent iron loading uniformity and precise composition control, enabling iron to be firmly attached to the biochar matrix, significantly enhancing the stability and consistency of material performance. Hydrothermal carbonization is conducted under mild conditions, featuring energy efficiency and environmental friendliness, while imparting the material with good pore structure and abundant functional groups, making it suitable for environmental scenarios with specific material performance requirements. Green synthesis methods adhere to environmental protection principles, endowing the material with good biocompatibility and offering broad prospects in fields such as biomedicine, with the sole need to improve synthesis efficiency. Ball milling is easy to operate and facilitates the mixing of biomass with iron sources, but it may damage the material structure to some extent. Chemical reduction can precisely control the form of iron, providing an effective approach for preparing high-performance and functionally specific materials, but the potential risks of chemical reagents must be properly addressed. In practical applications, synthesis strategies should be selected or optimized based on specific objectives, application scenarios, and cost considerations. If high efficiency, large-scale production, and relatively relaxed requirements for material uniformity are prioritized, one-step pyrolysis may be the optimal choice. Co-precipitation or chemical reduction methods may be more suitable for precise control of material properties. Green synthesis and hydrothermal carbonization are more beneficial for environmental friendliness and biocompatibility. As research progresses, synthesis strategies are expected to be further optimized and upgraded, achieving integration and complementarity, thereby driving the broader and deeper application of iron-based biochar materials in fields such as environmental remediation, energy storage, and biomedicine, and contributing to the resolution of various practical issues.

## 3. Application of Iron-Based Plant-Based Biochar in Fenton-like Applications

Iron-based biochar is widely used in applications similar to the Fenton method, and its performance can be optimized by adjusting the metal composition and composite materials. This section summarizes the performance of different iron-based plant-derived biochar catalysts in the remediation of organically polluted water bodies.

### 3.1. Iron-Based Monometallic Plant-Based Biochar

**Figure 3:**
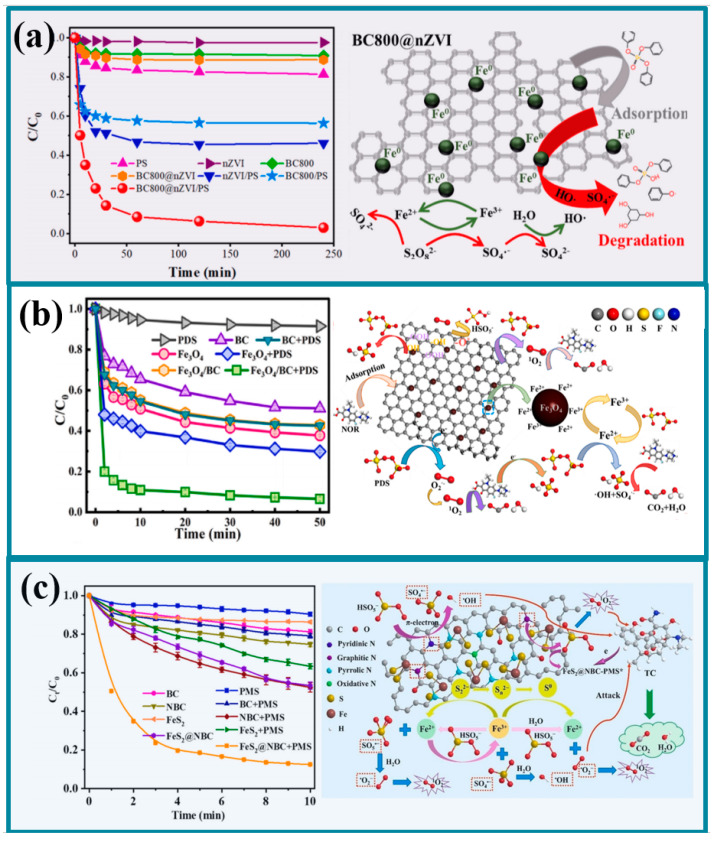
Application of different iron-based monometallic plant-based biochars in organic wastewater [45,57,58]. (**a**), Nanometer zero-valent iron-derived biochar from corn stalks (**b**), The process of active PDS degrading norfloxacin mediated by Fe_3_O_4_/BC (**c**), A nitro-nitrogen-doped biochar loaded with FeS_2_ was prepared, and the PMS degradation of tetracycline was achieved.

Iron is one of the most abundant elements on Earth. Based on iron, various nanomaterials and functional iron-based materials have been developed, including nanoscale zero-valent iron (nZVI), iron oxides (*e.g.*, *Fe_2_O_3_, Fe_3_O_4_*, *and FeOOH*), and iron sulfides (FeS). These materials have found widespread application in fields such as adsorption and catalysis [55,56]. Biochar possesses advantages such as a controllable two-dimensional layered structure, high specific surface area, high porosity, long-term stability, abundant oxygen-containing functional groups, low cost, and large adsorption capacity. By inhibiting the excessive agglomeration of metals, it can further generate a large number of active sites, making it widely used as a carrier for metal oxides. The application of iron-based materials based on plant-derived biochar in heterogeneous Fenton catalysis has attracted widespread attention. As depicted in Figure 3a, the corn stover-derived biochar loaded with nano zero-valent iron (BC800@nZVI) prepared by Li et al. [57] exhibited excellent performance for the degradation of triphenyl phosphate (TPhP) in water, with 96.9% of TPhP effectively removed within 4 h and the catalytic degradation kinetic rate as high as 0.0484 min^−1^. During the degradation process, BC has excellent specific surface area and pore structure, which can effectively adsorb TPhP, thereby promoting contact between ROS and pollutants and improving the utilization efficiency of ROS. PDS generates SO_4_^•−^ and ^•^OH by accepting electrons supplied by Fe^0^ and Fe^2+^. At the same time, Fe^0^ promotes the reduction between Fe^3+^ and Fe^2+^. Tian et al. [45] found that the presence of BC promoted the adsorption of norfloxacin and PDS during the degradation of norfloxacin by activated PDS by Fe_3_O_4_/BC, and revealed that the oxygen-containing functional groups on the surface of the material (-COOH and -OH) were the major contributors to the degradation of norfloxacin by comparing the X-ray photoelectron spectroscopy (XPS) of Fe_3_O_4_/BC before and after the reaction. Electron transfer mediators between PDS and the material, which promoted the generation of ROS and formed a synergistic effect with the active site iron (Figure 3b). Bao et al. [58] activated PMS degradation of tetracycline through the preparation of nitrogen-doped biochar loaded with FeS_2_, in which it was found that the graphitic nitrogen in the carbonaceous material dominated the generation of monoclinic oxygen (^1^O_2_) through the increase in the electron density of the carbon skeleton by promoting the generation of the non-radical reactions during the degradation process. Additionally, the lone pair of electrons on pyridine nitrogen activate PMS through π-electron transfer, reinforcing the radical process, while the S in FeS_2_ regenerates Fe^2+^ through a reduction reaction (Figure 3c).

### 3.2. Iron-Based Polymetallic Plant-Based Biochar

**Figure 4:**
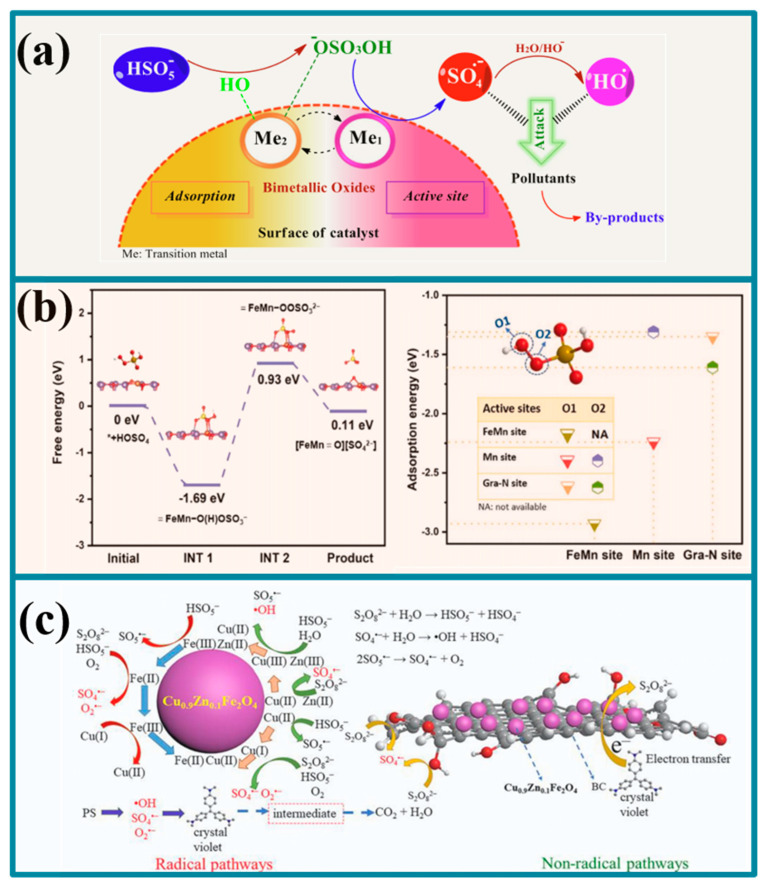
Application of different iron-based polymetallic plant-based biochars in organic wastewater [2,60,61]. (**a**), The bimetallic catalyst forms an adsorption-activation synergistic effect (**b**), Enhanced the mechanism of non-free radical pathways during the degradation process (**c**), The mechanism diagram for the generation of oxygen vacancies on the surface of CuFe_2_O_4_@BC materials due to zinc doping.

Monometallic catalysts are not suitable for practical applications due to their low activity, high cost, high metal ion release, and poor stability. The introduction of a second metal into an iron-based catalyst can effectively accelerate the redox cycle of Fe^2+^/Fe^3+^ and thus achieve continuous activation of the oxidant. Biochar-supported Fe-Mn composites (Fe-Mn@BC) prepared by Chen et al. [59] showed stronger activity relative to monometallic materials in the activation of a peroxynitrite-degrading aqueous solution in an acidic Red 88 system, attributed to the fact that Mn^2+^ promotes the reduction in Fe^3+^ to Fe^2+^, thus enhancing the activation of persulfate. Interestingly, Kohantorabi et al. [2] suggested that bimetallic catalysts can form an adsorption–activation synergy, specifically, Me_2_-OH, a highly oxidized metal center, produces ROS by adsorbing PMS and forming -OSO_3_OH, which is then activated by Me_1_ (Figure 4a). Similarly, He et al. [60] revealed the presence of -OSO_3_OH using DFT analysis and found that PMS becomes adsorbed on the FeMn@NBC surface as *HOSO_4_ and generates FeMn-O(H)OSO_3_^−^ complexes, which are further formed by FeMn-OOSO_3_^2−^ of the O-O bond is radicalized and dissociated, spontaneously generating the highly active species of high-valent bimetallic FeMn=O. More importantly, FeMn bimetallic sites have stronger adsorption capacity for PMS than single-metal Mn sites. By adsorbing the O1 site of PMS, the bimetallic sites on PMS release H atoms and e-oxidize to SO_5_^•−^, thereby enhancing the non-radical pathway during the degradation process (Figure 4b). Fan et al. [61] found that Zn doping could effectively enhance the generation of oxygen vacancies on the surface of the CuFe_2_O_4_@BC material, thus promoting the material-mediated direct electron transfer degradation between PDS and pollutants (Figure 4c). Therefore, multimetallic catalysts can not only promote intermetallic redox cycling, but also change the adsorption-oxidation mode of the oxidant, enriching the active sites of the catalysts and the degradation pathways of the pollutants.

### 3.3. Iron-Based Plant-Based Biochar Composites

With the increase in difficult-to-degrade organic pollutants, iron-based biochar catalysts are difficult to meet the demand, so researchers developed iron-based biochar composite catalysts. Wang et al. [62] prepared magnetic 2D/2D oxygen-doped graphitic carbon nitride/biochar (γ-Fe_2_O_3_/O-g-C_3_N_4_/BC) composites and used them for the activation of PMS for the degradation of emerging organic pollutants such as sulfamethoxazole, atrazine, phenol, nitrobenzene, carbamazepine, etc., in which sulfamethoxazole was removed by 100% in 240 min with a high mineralization rate of 62.3%. It was found that the excellent specific surface area and pore volume could provide more active sites for catalysis, and the close contact between the multilayer structure could help electron transfer. In addition, Ebrahimian et al. [63] also developed an iron and cobalt bimetallic composite multistage porous layered biochar catalyst, which exhibited a unique multilayer porous layered structure, reasonably reduced the agglomeration of metal nanoparticles, enhanced the dispersion and exposure of active sites, promoted the activation ability of PMS, and performed well in five cycles of tetracycline degradation with excellent stability.

### 3.4. Evaluation and Comparison of Different Catalyst Systems

Overall, the three catalyst types show distinct trade-offs in performance and practicality. Single-metal iron-based biochars can achieve high pollutant removal in lab settings (e.g., ~97% triphenyl phosphate degraded in 4 h), but they often suffer from limited activity and catalyst longevity under real-world conditions due to issues like metal leaching and deactivation [57]. By contrast, multi-metallic (polymetallic) iron–biochar catalysts generally exhibit higher degradation rates and more robust reusability, as the secondary metal synergistically accelerates the Fe^2+^/Fe^3+^ redox cycle and generates additional active sites. For example, an Fe–Mn co-doped biochar outperformed its Fe-only counterpart in dye oxidation, with Mn^2+^ continuously regenerating Fe^2+^ to sustain oxidant activation. This synergy not only boosts reactive radical production but also helps stabilize the catalyst and suppress iron leaching, improving long-term stability [60]. Composite biochar systems (integrating iron-loaded biochar with other functional materials) further enhance catalytic efficacy and durability, achieving near-complete contaminant removal with higher mineralization rates and maintaining activity over multiple use cycles. Their structured multi-component architecture [63] (e.g., 2D/2D carbon nitride–biochar hybrids or porous Fe–Co layered biochars) provides a high surface area and well-dispersed active phases, which promote pollutant access and electron transfer while minimizing nanoparticle agglomeration. However, these performance gains come with increasing preparation complexity and scalability challenges. Monometallic Fe–biochar catalysts are relatively straightforward to produce in bulk (often via one-step pyrolysis of biomass with an iron salt), whereas introducing additional metals or forming composites entails more elaborate, multi-step syntheses that can raise costs and reduce yield (for instance, gentle hydrothermal or “green” synthesis routes yield low biochar output, limiting large-scale production). In practical wastewater treatment, there is thus a balance to be struck: single-metal biochars offer simplicity and low cost, but multi-metal and composite catalysts deliver superior pollutant degradation and stability. The latter two systems appear more promising for treating recalcitrant pollutants and complex effluents due to their higher activity and reusability, provided their fabrication can be scaled up and issues like metal leaching are managed. Table 2 lists the comparison of the catalysts.

## 4. Application and Performance of Fe-Biochar Composites in Photo-Fenton Systems

Photo-Fenton systems that utilize Fe-based biochar catalysts have been applied to the degradation of a wide range of organic pollutants in both controlled and natural water matrices. Performance depends on iron speciation, biochar type, light source, and reaction conditions (pH, oxidant dose, catalyst loading) [64]. Recent studies have focused on optimizing the composition of Fe-biochar composites and integrating visible or solar-light activation to improve energy efficiency and environmental compatibility [65].

### 4.1. Light Source and Operational Conditions

Artificial light (UV-A, xenon lamp, 420–500 nm LEDs) is commonly used in laboratory studies to maintain consistent irradiance. Solar-driven photo-Fenton systems using Fe–biochar catalysts derived from agricultural residues (e.g., rice straw, sawdust) are gaining attention due to lower operational cost [66]. Catalyst performance is optimal at pH 3–5 for H_2_O_2_-based systems but can extend to near-neutral conditions (pH 6–7) when PMS is used or when Fe–N–C moieties are stabilized within graphitic biochar.

### 4.2. Photocatalytic Degradation of Emerging and Persistent Pollutants

Fe-biochar catalysts have been reported to degrade dyes (Congo red, methyl orange), pharmaceuticals (ciprofloxacin, naproxen), endocrine disruptors (bisphenol A), and pesticides (atrazine) under light-assisted Fenton conditions [67,68]. Removal efficiencies up to 95–100% have been achieved in 15–60 min using Fe–N–C and Fe–C_3_N_4_ catalysts at pollutant concentrations of 10–50 mg L^−1^ [69]. Visible-light photo-Fenton degradation of recalcitrant phenolic compounds and PFAS derivatives has also been reported when non-radical ^1^O_2_ pathways dominate in N-doped biochars [70].

### 4.3. Matrix Effects and Reusability

Matrix constituents such as NOM, HCO_3_^−^, and Cl^−^ can suppress radical activity but have less impact on non-radical ^1^O_2_ pathways. Stability testing shows that Fe leaching is usually <0.5 mg L^−1^ per cycle for well-anchored Fe–biochar catalysts. Reusability over 4–7 cycles has been demonstrated with <20% decrease in removal performance [71]. Fe encapsulation in carbon shells or immobilization within heteroatom-enriched domains enhances catalyst longevity.

Overall, Fe-biochar composites have demonstrated strong potential as multifunctional catalysts for light-assisted advanced oxidation processes, achieving high degradation efficiencies for a broad range of organic pollutants under UV, visible, and solar irradiation. Their performance is governed by the interplay between Fe speciation, carbon matrix structure, and operating conditions such as pH, oxidant type, and light wavelength. Monometallic Fe-biochars show good activity at low cost, while bimetallic and semiconductor-hybrid systems enable faster photoredox cycling, improved visible-light utilization, and reduced Fe leaching [69,72]. Non-radical ^1^O_2_ pathways in heteroatom-modified biochars further expand applicability to natural waters where radical scavenging is significant. Although laboratory-scale results consistently report removal efficiencies above 90% with acceptable metal release and multi-cycle reusability, large-scale application will require standardization of performance metrics, optimization of solar-driven reactor configurations, and techno-economic validation [70]. Continued development of structurally stable, low-leaching Fe-biochar catalysts and pilot-scale demonstrations will be essential for transition from proof-of-concept studies to practical water treatment systems.

## 5. Activation Mechanism

Persistent free radicals (PFRs), special structures (defective and graphitized structures) and oxygen-containing functional groups (-OH and -COOH) in pristine biochar catalysts are usually considered as active sites, whereas iron-based biochar materials add more active metal sites, which play an important role in the Fenton-like process [73]. The main Fenton-like mechanisms can be classified into free radical and non-free radical pathways (singlet oxygen, direct electron transfer, and high valence metals), and in this way, this section describes the catalytic oxidation process of active sites with oxidizing agents and explores the roles of the different reactive substances.

### 5.1. Radical Pathways

**Figure 5:**
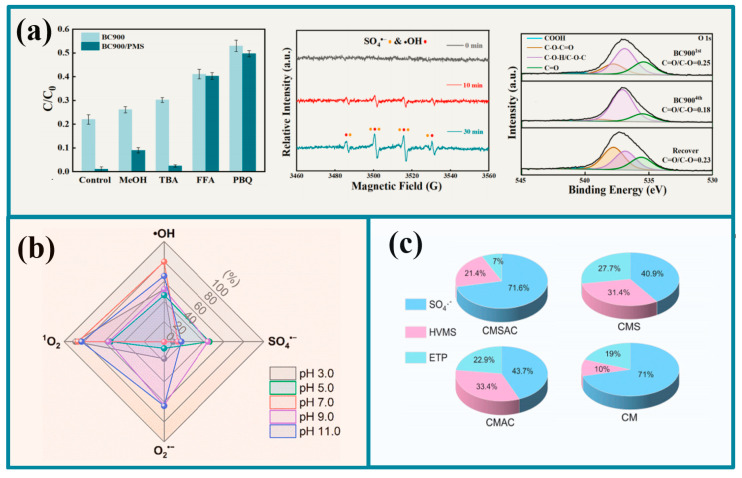
The comparison of quencher experimental results, EPR results and XPS before and after BC900 reaction in the BC900/PMS system (**a**), contribution of ROS to the degradation of ofloxacin under different pH conditions (**b**), and contribution of free radical and non-free radical pathways to the degradation of different contaminants (**c**) [76,78,87].

SO_4_^•−^ and ^•^OH are the main ROS of the free radical pathway, specifically generated through electron gain by oxidizing agents (Equations (1)–(4)). For pristine biochar, whose surface is rich in oxygen-containing functional groups, -OH and -COOH are usually considered as the active sites for SO_4_^•−^ and ^•^OH. In general, the role of SO_4_^•−^ and ^•^OH on pollutant degradation can be verified by analyzing the inhibition of pollutant degradation due to the high reaction rate constants of some free radical quenchers for ROS [74]. Zhang et al. [75] selected TBA (*k* ^•^OH = 5.2 × 10^10^ M^−1^·s^−1^) and MeOH (*k* SO_4_^•−^ = 2.5 × 10^7^ M^−1^·s^−1^) as ^•^OH and SO_4_^•−^ inhibitors. The effect of the inhibitor addition on pollutant removal was compared and it was found that ^•^OH and SO_4_^•−^ were involved in the degradation of pollutants and further detected by EPR. The characteristic peaks of ^•^OH and SO_4_^•−^ were subsequently examined by XPS for changes in the content of functional groups of the biochar material before and after use, thus verifying that the oxygen-containing functional groups (-OH and -COOH) of biochar are usually considered as the active sites of SO_4_^•−^ and ^•^OH active sites (Figure 5a). Zheng et al. [76] also found that PMS can form SO_5_^•−^ by losing electrons and also found that oxygen in the reaction system can generate superoxide anion radicals by gaining electrons (O_2_^•−^) and confirmed the presence of O_2_^•−^ using p-benzoquinone quenching experiments and EPR (Equation (5)). Typically, Fe^2+^ activation of PMS to form SO_5_^•−^ is thermodynamically infeasible (Fe^3+^/Fe^2^+ = 0.77 V, HSO_5_^−^/SO_5_^•−^ = 1.1 V) (Equation (6)). However, it relies on biochar’s unique persistent free radicals (PFRs) and carbon defects, which promote redox reactions between Fe^3+^/Fe^2+^ and enhance the production of free radicals [77]. Although all these free radicals contribute to the degradation of pollutants, there is a greater need to quantify these contributions, so Chen et al. [78] used kinetic modeling to analyze the reaction rate constants of different ROS in the system and calculated the rate constants of SO_4_^•−^, O_2_^•−^, and ^•^OH contributions to the degradation of ofloxacin under different pH conditions (Figure 5a).

O_2_ + e^−^ → O_2_^•−^ (5)

Fe^3+^ + HSO_5_^−^ →Fe^2+^ + SO_5_^•−^ + H^+^ (6)

O_2_^•−^ + ^•^OH → ^1^O_2_ + OH^−^ (7)

4SO_5_^•−^ + 2H_2_O → 4HSO_4_^−^ + 3^1^O_2_ (8)

### 5.2. Non-Radical Pathways

#### 5.2.1. ^1^O_2_ Pathway

In real aqueous environments, free radicals with strong oxidizing properties are less effective in degrading pollutants due to the presence of complex interfering substances such as natural organic matter, including SO_4_^2−^, Cl^−^, and HCO_3_^−^. In contrast, single linear oxygen (^1^O_2_) is considered to be more selective for pollutants [79]. A linear relationship was found between carbonyl content and catalytic activity of biochar. It has been reported that oxidants can be specifically adsorbed onto the active sites of carbon materials, and subsequently, ^1^O_2_ is reacted by these active sites (e.g., C=O and sp^2^-C) with PDS to degrade organic pollutants via the non-radical pathway [80]. Cheng et al. [26] also found that, among the various oxygen-containing functional groups of carbon nanotubes, the C=O group was the major active site that facilitated the activation of PDS for the oxidation of 2,4-dichloro phenol as the main active site. Furfuryl alcohol is commonly used as a ^1^O_2_ quencher due to its high reaction rate constant with ^1^O_2_ (k ^1^O_2_ = 1.2 × 10^8^ M^−1^·s^−1^), and similarly, 2,2,6,6-tetramethyl-4-piperidinone, as a ^1^O_2_ spin trapping reagent, can be detected as a set of intensities in EPR of 1:1:1 of TEMP-^1^O_2_ characteristic spectral lines [79,81,82]. It is noteworthy that SO_5_^−^ or O_2_^•−^ are also intermediates in the formation of ^1^O_2_ when using iron-based biochar as a Fenton-like catalyst [33,83]. And Chen et al. [78], in analyzing the contribution of ROS to pollutant degradation, found that ^1^O^2^ accounted for the major contribution to pollutant degradation in all pH = 3.0–9.0 conditions (Figure 5b).

#### 5.2.2. Direct Electron Transfer

The catalyst-mediated electron transfer process exhibits excellent selective oxidative properties and significantly avoids the adverse effects of common interferents in water. This process is achieved by the formation of surface-limited activated oxidant complexes on the iron-based biochar catalyst. In the absence of contaminants, the electron transfer process is slow. Once contaminants are present in the system, the electron transfer is rapidly initiated, leading to efficient degradation of the contaminants [84,85]. Generally speaking, researchers generally use substances with strong oxidizing properties to inhibit the degradation of pollutants by electron transfer in the system, and common electron inhibitors include Cr^6+^, ClO_4_^−^, etc. Wang et al. [86] found that PMS* complexes with high redox potentials were essential electron shuttles in the catalyst-mediated electron transfer process through electron transfer inhibition experiments and electrochemical tests. Bimetallic complexes and carbon materials reduced the transfer resistance between the catalyst and oxidant and promoted the electron transfer process in degrading pollutants. In addition, the presence of BC not only promotes the degradation of pollutants by direct electron transfer, but also indirectly promotes the degradation of pollutants by enhancing the electron transfer between the composite and the oxidant [87]. Interestingly, Hu et al. [88] calculated the contribution of electron transfer to pollutant degradation by the exclusion method; however, in the case of a wide variety and complexity of active substances, the feasibility of this method is low, and a more intuitive method needs to be further investigated (Figure 5c).

#### 5.2.3. High-Value Metals

In addition to the common active substances in Fenton-like, high-valent metal, represented by Fe^4+^ play a key role in pollutant degradation. Among them, Fe4+ can be obtained from Fe^2+^ activated oxidants (Equations (9)–(12)). It has been reported that dimethyl sulfoxide (DMSO) is commonly used as a quencher for high-valent metals and that high-valent metals can react with methyl phenyl sulfoxide (PMSO) to form specific methyl phenyl sulfone (PMSO_2_), so the presence of high-valent metals has also been verified by determining the concentrations of PMSO and PMSO_2_ [89,90]. Pei et al. [91] found that 5,5-dimethyl-1-pyrroline-N-oxide (DMPO) can react with high valence metals to form 5,5-dimethylpyrroline-(2)-oxy-(1) adducts (DMPOX) with 1:2:1:2:1:2:2:1 peak shapes, which further suggests the presence of high valence metals. Li et al. [92] demonstrated that the high valence metal species have long lifetimes (7–10^−1^ s, a steady-state concentration of 10^−8^ M) and are less sensitive to the removal of non-target substrates such as natural organic matter.

Fe^2+^ + O_3_ → FeO^2+^ + O_2_ k = 8.3 × 10^5^ M^−1^·s^−1^ (9)

Fe^2+^ + H_2_O_2_ → FeO_2_+ + H_2_O k = 7.6 × 10^1^ M^−1^·s^−1^ (10)

Fe^2+^ + HSO_5_^−^ → FeO^2+^ + SO_4_^2−^ + H^+^ k = 2.2 × 10^4^ M^−1^·s^−1^ (11)

Fe^2+^ + S_2_O_8_^2−^ → FeO^2+^ + 2SO_4_^2−^ + 2H^+^ k = 2.0 × 10^1^ M^−1^·s^−1^ (12)

## 6. Challenges and Mitigation Strategies of Iron-Based Biochar Catalysts in Industrial-Scale Applications

### 6.1. Cost–Benefit and Scale-Up Assessment

Although Fe-based biochar catalysts demonstrate strong reactivity and reusability in laboratory conditions, their large-scale feasibility depends critically on the balance between catalytic efficiency and economic viability [93]. The cost structure of these materials can be analyzed through the combined contribution of feedstock procurement, energy input, chemical reagents, and recovery operations. A practical approach to cost evaluation considers the unit catalyst cost (UC):

(13) $$UC=\frac{C_{feedstock}+C_{reagents}+C_{energy}+C_{labor}+C_{capital}}{Y}$$

where Y is the yield (kg catalyst/·kg feedstock). Comparative assessments show that pyrolysis temperatures above 700 °C increase performance but disproportionately raise energy demand [94]. Therefore, optimization at moderate temperatures (500–600 °C) is often recommended for a balance of reactivity and cost efficiency.

At industrial scale, continuous pyrolysis and waste-heat recovery systems can reduce production costs by up to 40% compared with batch systems [95]. Table 3 outlines a framework for cost assessment and comparison with conventional oxidation technologies. Utilize low-cost or waste biomass (e.g., agricultural residues, invasive plants) as feedstock to reduce material costs. Optimize production processes (e.g., one-step pyrolysis or hydrothermal methods) for energy efficiency and larger throughput to achieve economies of scale [96]. Conduct cost–benefit analyses to balance catalyst expense against pollutant removal efficiency, guiding economic optimization [93].

### 6.2. Metal Dissolution and Secondary Pollution Control

A major environmental concern during an AOPs operation is metal leaching from Fe-based biochar, which can introduce secondary pollution if uncontrolled [97]. Dissolved Fe^2+^/Fe^3+^ may catalyze homogeneous reactions but accumulate as sludge after repeated cycles, compromising both water quality and catalyst reusability. Reported leaching levels vary widely, from <0.5 mg L^−1^ in well-stabilized Fe_3_O_4_–biochar composites to > 10 mg L^−1^ in uncoated Fe-impregnated materials under acidic conditions [98].

Leaching behavior depends on pH, surface speciation, and synthesis route. Strategies to mitigate dissolution include [99]:

1. Encapsulation of Fe nanoparticles within graphitic carbon layers to shield against acid attack;
2. Formation of stable oxides (Fe_3_O_4_, γ-Fe_2_O_3_) rather than free FeO;
3. Sulfidation to produce FeS_x_ species with low solubility;
4. Surface complexation with oxygen- or nitrogen-containing functional groups to strengthen metal–support interactions (MSI);
5. Post-treatment annealing at 500–600 °C to enhance crystallinity and reduce labile Fe sites.

### 6.3. Applicability to Real Water Matrices

Most AOPs studies are conducted in idealized, deionized water; however, natural water matrices contain ions and organic matter that significantly alter reactive oxygen species (ROS) dynamics [100]. Bicarbonate and chloride ions act as radical scavengers, while natural organic matter (NOM) competes for oxidants, reducing pollutant degradation efficiency [101]. Therefore, matrix complexity must be explicitly considered when assessing the practical performance of Fe-based biochar catalysts.

To bridge laboratory and field conditions, we recommend a three-tier evaluation protocol:

1. Baseline tests in deionized water to quantify intrinsic catalytic kinetics.
2. Simulated water matrices containing common inorganic ions (Cl^−^ ≈ 50 mg L^−1^, HCO_3_^−^ ≈ 200 mg L^−1^, Ca^2+^ ≈ 100 mg L^−1^) and NOM (5–10 mg L^−1^ TOC) to assess interference.
3. Actual water samples (e.g., river water, secondary effluent) to verify field applicability.

### 6.4. Toxicity and Transformation Product Assessment

While high pollutant removal rates are frequently reported, the formation and persistence of toxic intermediates remain insufficiently investigated [102]. A comprehensive evaluation of transformation products is essential to ensure that AOPs achieve true mineralization rather than partial oxidation.

Key analytical approaches include:

1. Identification of intermediates via GC–MS or LC–MS coupled with accurate mass spectrometry (MS/MS).
2. Quantification of total organic carbon (TOC) to evaluate mineralization.
3. Ecotoxicity assays such as Daphnia magna immobilization, Pseudokirchneriella subcapitata algal growth inhibition, or Vibrio fischeri luminescence inhibition (OECD standard tests).
4. Computational toxicology tools (QSAR models, ECOSAR) for predicting the potential hazards of intermediates.

Reported results suggest that although Fe-biochar systems achieve > 90% parent pollutant degradation, TOC removal often remains below 60%, implying incomplete mineralization [102]. Toxicity assays sometimes show transient increases in acute toxicity during the initial oxidation stages, followed by decline after prolonged treatment [103]. These findings emphasize the need for integrated degradation-toxicity assessments.

### 6.5. Catalyst Stability and Longevity

The operational stability and reusability of Fe-based biochar catalysts are critical parameters that determine their long-term applicability in real-world water treatment systems. While high initial degradation efficiencies are often reported, catalyst performance typically declines over multiple reaction cycles due to iron leaching, surface fouling, or structural degradation [104]. For example, low-valence Fe(II) species can progressively dissolve into the liquid phase under acidic or highly oxidative conditions, resulting in secondary metal pollution and loss of active sites. Additionally, residual organic matter, radical scavengers, and carbonate or chloride ions in natural waters can adsorb or react on catalyst surfaces, inhibiting active sites and suppressing ROS generation [105]. To overcome these challenges, several stabilization strategies have been proposed, including graphitic encapsulation of iron nanoparticles, core–shell architectures, sulfidation of Fe phases, and anchoring Fe oxides within heteroatom-doped carbon matrices, which together can significantly reduce dissolution and improve cycling durability. Standardized leaching tests (e.g., cumulative Fe release after ≥5 cycles) and analysis of catalyst crystallinity, particle morphology, and surface chemistry after reuse (via XRD, XPS, TEM) are strongly recommended to quantify stability. These evaluations are essential not only for mechanistic insight but for achieving compliance with effluent quality standards during practical implementation. Table 4 lists the key factors that affect the stability and reusability of iron-based biochar catalysts in advanced oxidation processes.

Future research should implement a mass-balance-based pathway evaluation combining quantitative intermediate profiling and toxicity endpoints to discern whether observed byproducts are persistent or biodegradable. Standardized reporting of both chemical and biological parameters will strengthen the environmental relevance of Fe-based biochar AOP studies.

## 7. Conclusions and Perspective

Iron-based biochar holds great promise as a catalyst for removing organic pollutants, thanks to its ease of synthesis, excellent catalytic performance, and reusability. The unique structure and composition of plant-based biomass make it an ideal high-quality carbon source for biochar production, while pyrolysis conditions significantly influence biochar performance, offering a variety of synthesis strategies for material preparation. In Fenton-like applications, iron-based plant-derived biochar in single-metal, multi-metal, and composite forms has demonstrated the ability to degrade organic pollutants, with its activation mechanisms involving both radical and non-radical pathways. Looking ahead, future research on Fe-biochar systems should focus on the following priorities:

1. Durability and leaching control: Improve metal–support interaction via structural encapsulation or sulfidation to enable long-term operation with Fe release below regulatory limits and consistent catalytic activity over multiple reuse cycles.
2. Standardized performance metrics: Establish reporting guidelines that include Fe leaching per cycle, photon-normalized kinetics, total organic carbon (TOC) removal, and toxicity evaluation of intermediates to enable fair comparison across studies.
3. Cost–benefit assessment: To explore the economic cost of iron-based plant biochar in organic wastewater treatment, especially in large-scale production scenarios, and to weigh the pollutant removal efficiency against the cost, so as to help promote its practical application.
4. Metal dissolution control: Closely monitor the metal dissolution condition in the catalyst use, strictly control it according to the emission standard, prevent secondary pollution, and ensure the environmental treatment effect and environmental safety.
5. Research on the suitability of actual water bodies: In view of the complexity of the actual water body environment, strengthen the application research in natural water bodies, precisely analyze the impact of actual water body factors on the treatment effect, and enhance the practicality of the technology.
6. Enhanced toxicity risk assessment: Emphasize the assessment of toxicity and biological hazards of intermediate products, fill the gaps in existing research, avoid secondary pollution and biological toxicity caused by more toxic intermediate products in the treatment process, and safeguard ecological safety.

## Figures and Tables

**Table 1:** Various methods of synthesizing iron-based plant-based biochar and their advantages and disadvantages.

Preparation Method	Advantages	Disadvantages
One-step pyrolysis	Easy to operate and highly controllable	Limitations, high equipment requirements, more impurities
Co-precipitation	Highly controllable and homogeneous active site	More impurities, longer time spent
Hydrothermal carbonization	Gentle and environmentally friendly conditions	High equipment requirements and low yield
Green synthesis	Environmentally friendly, highly sustainable, low toxicity	Low throughput and poor repeatability
Ball milling	Easy handling, homogeneous active site	Poor controllability, easy to destroy the original structure
Chemical reduction	Material homogeneity, controllability, efficient synthesis	High cost of reducing agents, environmental hazards

**Table 2:** Representative Fe-biochar catalyst classes: efficiency, scalability, full-scale applicability [58,63].

Catalyst Class	Typical Strengths (Bench Scale)	Practical Limitations	Scalability and Field Applicability (Assessment)
Monometallic Fe–biochar	High removal (>90%) for many organics; low cost; simple one-step pyrolysis routes	Fe leaching at low pH; faster decay in real waters; sometimes lower mineralization	High scalability (simple synthesis), moderate field readiness if leaching is controlled and non-radical pathways are present
Bimetallic/multimetallic Fe–M–biochar	Faster Fe(III)/Fe(II) cycling; lower oxidant demand; better cyclic stability	More complex synthesis; dopant cost; need to monitor secondary metals	Moderate–high scalability (co-pyrolysis/impregnation possible), good field potential with stable Fe/M binding and reuse ≥5 cycles
Composites	Highest activity/mineralization; non-radical selectivity; strong matrix tolerance	Multi-step fabrication; yield and cost penalties	Moderate scalability (requires process optimization), high field promise for recalcitrant pollutants/complex matrices

**Table 3:** Framework for cost and practicality evaluation of Fe-based biochar catalysts [94].

Component	Typical Range or Consideration	Economic Implication	Recommended Optimization
Feedstock	Agricultural or forestry waste, 0–50 USD t^−1^	Low cost; supply chain stability critical	Prioritize local biomass sources
Energy input	Pyrolysis 400–800 °C	Dominant cost contributor	Moderate temperature, waste-heat recovery
Iron precursor	FeCl_3_, Fe(NO_3_)_3_, FeSO_4_ (0.2–1.0 USD kg^−1^)	Moderate; scalable	Recycle iron-containing wastewater
Activation agents	KOH, H_3_PO_4_, etc.	Costly and corrosive	Explore physical activation or one-pot synthesis
Catalyst recovery	Magnetic separation or filtration	Low cost per reuse	Design for magnetic recyclability
Comparison with homogeneous Fenton	Requires pH ≈ 3, generates sludge	Biochar avoids excess sludge	Emphasize stability and reusability

**Table 4:** Key factors influencing stability and reusability of Fe-based biochar catalysts in AOPs.

Stability Factor	Underlying Cause	Evaluation Method	Mitigation Strategy
Iron leaching	Weak Fe–C interaction; acidic pH; valence cycling	ICP-OES/MS for dissolved Fe per cycle; total Fe elution over ≥5 cycles	Graphitic encapsulation; Fe_3_O_4_/Fe_2_O_3_ crystallization; sulfidation (FeS_x_ stabilization)
Surface fouling	Adsorption of NOM, carbonate, chloride, or reaction intermediates	FTIR/XPS before/after cycles; TGA for carbonaceous deposits	Pre-filtration; periodic regeneration
Structural degradation	Breakage of carbon matrix under mechanical or chemical stress	SEM/TEM imaging after reuse; BET analysis	Pelletization; binder; magnetic recovery to limit abrasion
Oxidant overuse	Excess PMS/H_2_O_2_ generating ROS attack on catalyst itself	Adsorption capacity/reduction in BET area over time	Optimize oxidant dosage; promote non-radical pathways
Redox fatigue	Fe(III)/Fe(II) cycling imbalance; insufficient electron supply	XPS Fe 2p or Fe K-edge XANES before/after cycles	Introduce electron mediators
Mechanical attrition	Stirring, fluidization, abrasion losses	Mass balance; size distribution analysis; wet sieving	Use of magnetic Fe-biochar for rapid collection; integration into fixed-bed reactors
pH instability	Operating at low or fluctuating pH	Measure pH trend during cycles; correlate with Fe release	Design for neutral-pH operation (photo-Fenton, non-radical pathways)

## Data Availability

The datasets used and analyzed in this study are available from the corresponding author upon reasonable request.
